# Influence of Biodegradable Release Oils on the Physical and Mechanical Properties of Light-Colored Architectural Concrete

**DOI:** 10.3390/ma14164630

**Published:** 2021-08-17

**Authors:** Danuta Barnat-Hunek, Małgorzata Szafraniec

**Affiliations:** Faculty of Civil Engineering and Architecture, Lublin University of Technology, Nadbystrzycka 40, 20-618 Lublin, Poland; d.barnat-hunek@pollub.pl

**Keywords:** biodegradable release oils, light-colored concrete, contact angle, vapor permeability, adhesion

## Abstract

In the article, unique formulations of biodegradable, non-toxic, edible oil-based release oils were developed and tested on architectural concrete. The produced agents have physicochemical properties similar to diesel fuel, but at the same time, are renewable and biodegradable products. An ultrasound was used to properly combine the liquid phase of edible oil and the liquid phase of glyceryl trioleate and/or water. Based on the PN-B-19305 standard, seven-component configurations were designed and then tested. The wettability of the concrete was determined by contact angle (CA) analysis. After the application of the formulations produced, the architectural concrete still had good wettability. The vapor permeability test showed that the tested release agents did not inhibit water vapor diffusion from the tested samples. The O65G35 (65% unique edible oil formula and 35% glyceryl trioleate) concrete had the best absorption. In this case, the CA was 56° after oil application and 46° before. The new agents did not impair the adhesion of the plaster to concrete. The O90W10 concrete showed the best adhesion of plasters made with it—51.9 kN/m^2^. The study also showed that the concrete surface had excellent paint absorption despite the use of release agents. The architectural concrete was evenly covered with paint without any problem. There were no difficulties in applying it, e.g., greasy places preventing the concrete from being coated with emulsion. The suitability of the produced release oils for lightweight architectural concrete structures intended for facades was confirmed. The best results were obtained after using formulations O65T35 and O90W10.

## 1. Introduction

A characteristic feature of concrete as a construction material is the formation of elements of practically any shape. In the case of precast elements, this is performed in suitably prepared molds, usually reusable. In monolithic structures, modular or individual formwork is used, which generally can also be used again. The forming process consists of tightly filling the mold with concrete mix. Once the concrete is placed in the mold, the chemical processes of setting and hardening begin. The cement used as a binder in concrete due to transformations and reactions turns into, among other things, calcium hydroxide, which is the cause of a highly alkaline environment. As a strong alkali, it has corrosive properties which are harmful if they come into contact with the skin, and it also affects the surface reaction between the mold material and the concrete. The hardened concrete will stick firmly to the mold and cause two undesirable effects during demolding—damage to the surface of the molded concrete element or damage to the mold (formwork). Damage to the surface of the concrete generally needs to be repaired for the sake of the structure’s design life. In concretes with expected visual parameters (e.g., architectural concrete), it may disqualify it entirely and lead to the demolition of the element and reconstruction. The only way to avoid problems remains to use an adequately selected release agent designed to minimize cohesion forces between contacting materials.

Sources of knowledge such as [1,2,3,4,5,6,7] on architectural concrete projects help clarify the general requirements, execution, evaluation, and acceptance of architectural concrete structures and elements. Due to the lack of regulations and standards for architectural concrete, the specifications usually consider the findings and requirements for concrete surface quality contained in German guidelines [4].

Release agents also are not covered by standard requirements, as in concrete admixtures or concrete additives, for example. However, they can be classified in a collaborative group of products, so-called construction chemicals. In the PN-EN 13,670 standard [8]—execution of concrete structures, in chapter 5.2.2, two basic requirements for release agents are formulated. Almost any substance that eliminates or reduces adhesion can be used as a release agent if it is not harmful to the concrete or formwork. The most commonly used preparations as release agents are oily substances of mineral or vegetable origin, usually modified with additives improving their effect or application. They may additionally contain diluents or occur in the form of water emulsions. More often, preparations in pastes using synthetic waxes, paraffin, or resins are used. Moreover, an important property expected from modern release agents is their biodegradability.

Diesel oils are often used as release agents. The disadvantages of diesel oils are toxicity related to their composition of harmful aromatic hydrocarbons, flammability (class II), unpleasant smell, and aggressiveness towards the environment. Even more destructive are lubricating oils and used oils that should not be used for these purposes due to their carcinogenic effect. For these reasons, in most industrialized countries with highly ecologically sensitive societies, there have been strong trends toward the search for molding oils that are harmless and compatible with the environment. In response to these trends and demands, unique formulations of biodegradable, non-toxic oils with reduced human and environmental impact were developed in this paper.

Release agents can be distinguished in three types: directly applied as oil, in the form of water-oil emulsions, and a gel. Baty and Reynolds [9] divided mold release agents into two categories–barrier release agents (non-reactive or passive) and reactive release agents (chemically active). Barrier release agents create a physical barrier between the mold and the concrete. In contrast, reactive agents contain an active ingredient that chemically combines with calcium (found in lime) in the fresh cement paste. The chemical reaction between the calcium and the release agent prevents a thin surface layer from forming on the concrete. The chemically active ingredient in the release agent—fatty acid, allows the formwork to be released from the concrete.

Fatty acids can be obtained by modifying vegetable oils such as canola oil, sunflower oil, coconut oil, or soybean oil by separating them into glycerol and the fatty acids themselves. A subsequent combination of these acids with another suitable alcohol gives fatty acid esters, which can then be emulsified in water [10]. Most barrier-type release agents are not recommended because creating a barrier between the mold and the fresh concrete requires such intensive use of these oils that holes and stains are often made (not to mention pollution problems) [11].

Baty and Reynolds [9] also suggest that barrier release agents are not the best option if a good quality surface finish for architectural concrete is required. Correctly formulated and applied, reactive type release agents can produce fewer holes, stains, and surface irregularities. Depending on the brand, they can remain on the mold for weeks without reapplication [10]. Reactive release agents can be divided into two further subcategories: mineral oil-based release agents and vegetable oil-based release agents. According to Djelal et al. [12], mineral oil-based release agents are being replaced by vegetable oil-based release agents because the latter are less harmful to the environment, especially if accidentally spilled on site.

Vegetable oil-based release agents have been marketed in Europe for nearly 40 years, and many successful companies have already proved their use. However, initial poor technical performance, low prices of mineral oil-based products, and lack of awareness of the environmental consequences, among other reasons, are responsible for the current minimal market share of this type of oil [10]. In the Polish literature, only one item was found concerning the use of higher fatty acids (FAME) to produce release oil [13]. It shows that it is reasonable to use FAME for the production of the release agent. Different variants of vegetable oil-based release agents can be distinguished: pure vegetable oils, fatty acid esters (ester oils), and emulsions of fatty acid esters or pure vegetable oils in water (emulsions). In terms of biodegradability, these products rank high among the release agents, including mineral oils with VOCs, mineral oils without VOCs, and biodegradable mineral oils. However, it should be noted that the biodegradability of vegetable oil-based release agents can be impaired by using additives such as emulsifiers, antifreeze additives, corrosion inhibitors, antioxidants, and others.

Biodegradability is one of the stringent environmental requirements that new generation release agents must meet. The ingredients used for the production of biodegradable release agents should absolutely meet strictly defined environmental requirements. For European countries, the most common needs are those adopted in Germany in the 1970s with the “Blue Angel” eco-label regulation. These guidelines have successfully introduced new technologies and are still used to modify lubricant compositions, create modern products with ecotoxicological properties, and limit lubricants’ impact on the environment.

Thus far, the classification of release agents has not been developed by the International Organization for Standardization (ISO). The withdrawn standard PN-B-19305 [14]—“Release agents for steel molds used in the production of elements from aggregate and cellular concrete”, classifies the release agents for concrete as emulsion agents (E) and oil-based agents (O). This standard distinguishes between two types of oils depending on the kind of concrete formed: cellular (L) and aggregate (K). A number of test methods and requirements essential for the effectiveness of mold release agents are provided in the withdrawn PN-B-19305 standard [14].

The primary outcome of this research is to evaluate the use of biodegradable release oils and their effects on the physical and mechanical properties of light-colored architectural concrete.

## 2. Materials and Methods

### 2.1. Materials

Release oils tests were conducted on architectural concrete with white CEM I 52.5 R cement with high color purity requirements.

Materials used to make the concrete mix:

– Portland cement CEM I 52.5R, with the following specifications: a specific surface area of 4080 cm^2^/g, beginning of binding 140 min, end 170 min; compressive strength after two days 36.0 MPa and after 28 days 56.6 MPa. Loss on ignition 2.1%, whiteness 75%, insoluble residue 0.43%, Cl content 0.06%, SO_3_ content 3.82%, volume change 0.8 mm [15]. Tests of Portland cement CEM I 52.5 R were conducted following EN 197-1 standard [16]. Portland cement CEM I 52.5 R is from a cement plant in Chełm, Poland (CEMEX Polska sp. z o. o.);
– Quartz sand (0–2 mm). The utilized sand is characterized by the following chemical composition: SiO_2_, Al_2_O_3_, Fe_2_O_3_, and CaO (95.3%, 1.9%, 0.7%, and 0.35%, respectively). The chemical composition of the quartz sand and the coarse aggregate was obtained using a scanning electron microscope (SEM)—Quanta FEG 250. Quartz sand is characterized by specific gravity equal to 2650 kg/m^3^, water absorption amounting to 1.2%, and moisture reaching 0.16% [15];
– Coarse aggregate—natural gravel quartz (2–8 mm) from light sandstone and minerals in gravel grains came from plutonic rocks, i.e., orthoclase and albite, minerals in grains came from sedimentary rocks—calcite, illite dolomite. The bulk density of sand and gravel used was the same, i.e., 2.65 kg/dm^3^;
– Superplasticizer was used in order to reduce the amount of water used in the production process. It is a highly liquefying agent based on polycarboxylates with a density of 1.06 ± 0.02 g∙cm^−3^ and pH 1–5. By using a polycarboxylic ether-based superplasticizer, a comparable consistency was obtained for all concrete samples.

The dry ingredients were slowly mixed in a mixer for 2 min. Then water with superplasticizer was slowly and gradually added to the mixture to achieve a thorough combination and avoid segregation of the mixture components. Mixing was continued for 5 min. The concrete mix was compacted in the molds on a vibrating table at a vibration frequency of 50 Hz to achieve complete compaction without segregation and release of cement laitance. After removing the mold, the samples were stored in water at 20 ± 5 °C for 28 days under laboratory conditions. The made concrete slump test for concrete mixture indicated 12 ± 2 cm, which reflects consistency class S3 [17].

The composition of the light-colored architectural concrete was as follows: Portland cement CEM I 52.5 R—374 kg/m^3^; coarse aggregate 2–8 mm—1156 kg/m^3^; quartz sand 0–2 mm—770 kg/m^3^; superplasticizer—3.3 kg/m^3^; water—112.2 L/m^3^; w/c ratio = 0.3.

Following seven release agents configurations were prepared in the study:

– O65G35—65% bioecobase and 35% bioecobase-o;
– O65G33W2—65% bioecobase, 33% bioecobase-o, and 2% water;
– O65G31W4—65% bioecobase, 31% bioecobase-o, and 4% water;
– O65G29W6—65% bioecobase, 29% bioecobase-o, and 6% water;
– O70W30—70% bioecobase and 30% water;
– O80W20—80% bioecobase and 20% water;
– O90W10—90% bioecobase and 10% water.

The method for the preparation of bioecobase and bioecobase-o includes the steps of heating inedible (formed as a result of improper storage, out-of-date, extraction, post-frying) vegetable oils (i.e., rapeseed, sunflower, soybean), preparing a catalytic mixture from methanol and potassium hydroxide, causing a reaction by combining the heated oil with the catalytic mix, resulting in a post-reaction combination.

Methods of producing bioecobase, bioecobase-o, and mold release agents are the subject of patent application number P.437754 dated 30-04-2021. The individual components were mixed using a slow-speed mixer for 5 min to combine thoroughly.

### 2.2. Methods

The general characteristics of the concrete were obtained from tests: volumetric density according to EN 12390-7 standard [18], compressive strength according to EN 12390-3 standard [19], flexural strength during bending according to EN 12390-5 standard [20]. A testing program for mold release agents was proposed based on the PN-B-19305 standard [14].

Density and viscosity of the release agent: the viscosity coefficient was determined by the Stokes method [21] at the room temperature of 22.5 °C. Six measurements were taken as the authoritative number. Storage temperature: the study was conducted in a climate chamber to demonstrate the effect of low temperatures on the aggregate state of the new release agents. A temperature of +5 °C was set in the chamber, and the release agent samples were stored in the chamber for 12 h. After the test was completed, the temperature was lowered in 2 °C increments until −14 °C.

Biodegradability: Biodegradability tests were performed under the conditions described in CEC Method L-33-T-82 [22,23]. According to the CEC methodology, 150 cm^3^ of 50 mg/dm^3^ solution were prepared for testing, into which 1 cm^3^ of inoculum containing more than 107 CFU/cm^3^ (active cells/cm^3^) were introduced each. The inoculum used for testing was obtained from municipal wastewater after mechanical treatment. This test determined the decrease in hydrocarbon concentration in a sample containing the test substance in a mineral medium inoculated with microorganisms. The release agent was introduced into the experimental vessels in carbon tetrachloride. The absorption maximum of CH_3_–CH_2_ bonds at 2930 cm^−1^ was determined in the obtained extracts. The concentration of hydrocarbons was determined spectrophotometrically by IR (NICOLET 380 FT IR spectrophotometer) after saponification of the samples by ultrasound and extraction with carbon tetrachloride. The determination of IR absorption was performed after 7 and 21 days of the experiment. The test allows the substance to be classified as readily biodegradable if it achieves, within 28 days, the required level of biodegradation of 70% for the determination of dissolved organic carbon and 60% for the determination of Biochemical Oxygen Demand or CO_2_ produced.

The effect of release agents on concrete surface quality: greasiness of concrete surface, color change of concrete surface, streaking (visual method). Influence of the release agents on the mold quality: adhesion—checking the effectiveness of the release agent. According to paragraph 2.3.2 of the PN-B-19305 [14] standard, the release agent should not cause a change in the color of the concrete (stains, crystalline efflorescence) on any of the tested samples. A set of molds was prepared to perform the tests:

– steel rectangular with dimensions 40 mm × 40 mm × 160 mm;
– plastic cubes with dimensions of 100 mm × 100 mm × 100 mm;
– wooden furniture board 50 mm × 300 mm × 300 mm.

The molds were thoroughly cleaned, and then their walls were coated with a release agent.

The effect of release agents on the characteristics and durability of concrete: the water absorption coefficient A_w_ (kg/(m^2^ s^0.5^)) due to capillary rise was performed according to PN-EN 1015-18 [24] standard on rectangular-shaped specimens with dimensions of 40 mm × 40 mm × 160 mm. Wettability measurement (water contact angle CA) characterizing the liquid drop was measured on a research stand, comprising a goniometer and a camera used for capturing the image of a drop put onto the surface of a sample. The contact angle analysis was conducted with distilled water using a goniometer (Data Physics Instruments GmbH, Filderstadt, Germany). The constant volumes of liquid drops approximated 2 mm^3^ and were applied onto the sample via micropipette. Five drops were applied to each sample, given the heterogeneity of the material. The measurements were conducted at the temperature approximating 22.5 °C [25]. The water vapor diffusion test was conducted to see if the release oils have a negative effect on the movement of water vapor through the concrete sample. After determining the water absorption coefficient due to capillary rise, the samples were pulled out of the water and surface dried. The specimens were left to evaporate in the laboratory at 20 ± 5 °C. The measurement was performed successively after 1, 3, and 7 days of noting the successive decreases in material weight on the analytical balance.

The adhesive strength of the hardened plasters on concrete samples was tested in accordance with EN 1015-12 standard [26]. Adhesion was defined as the maximum tensile stress induced by a peel load applied perpendicular to the mortar surface. The metal, round plates with a diameter of 50 mm were glued on their surface with an epoxy resin adhesive. The plates were pulled out using an extractometer DTEpico-VCH 2500 (Dynatest) as the bond had set. The arithmetic mean of five results was taken as the value. Effect of release agent on the ability to coat the concrete with paint: paint absorbency, uniform paint coverage of concrete.

## 3. Results

### 3.1. The Basic Characteristics of the Concrete

The primary characteristics of concrete that are not addressed in this study are volumetric density 2295 kg/m^3^, compressive strength f_c,cube#150_ = 75.4 MPa, and flexural strength during bending 8.2 MPa. The mechanical strength values (compressive and flexural) were obtained after 28 days.

### 3.2. Release Agent Viscosity and Release Agent Density

**Figure 1:**
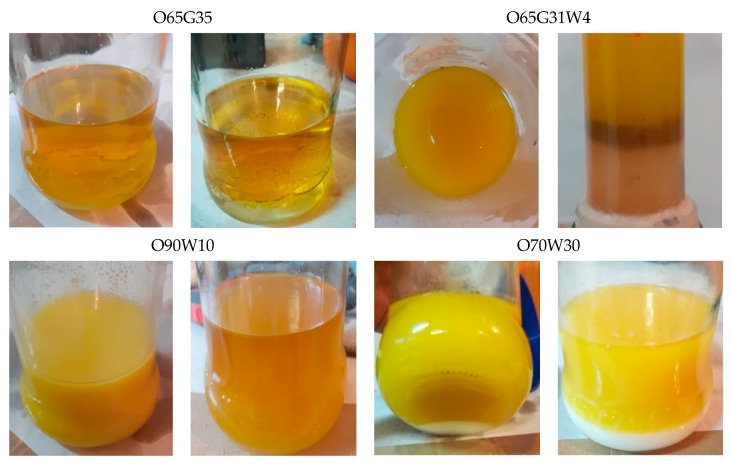
The appearance of selected mold oils immediately after mixing and after 3 h.

Figure 1 shows the condition of the new release agents immediately after mixing and after 3 h. The results of the viscosity coefficient η and volume density of the mold release agents are summarized in Table 1.

The tested newly produced agents have a density of 1.0–1.1 g/cm^3^. Water at a temperature of about 1 °C has a similar density. Table 1 also shows that the formulations produced have dynamic viscosities in the range of 0.022 to 0.037 Pa·s and kinematic viscosities in the range of 25.30 to 33.56 mm^2^/s. The highest values of both dynamic and kinematic viscosity were characteristic for the preparation of O65G31W4.

### 3.3. Storage Temperature

The results obtained are summarized in Table 2.

The best parameters were obtained for formulation O65G35 because even at a temperature of −14 °C, it remained liquid and did not freeze. Preparations with water admixture froze depending on the amount of this admixture (from 0 to −10 °C).

### 3.4. Biodegradability

The biodegradability results are shown in Table 3. Biodegradability was determined based on the concentration of hydrocarbons was determined spectrophotometrically by using the FT IR test.

The test showed that all the oils obtained can be classified as readily biodegradable because, after 21 days, the level of biodegradation was between 98 and 100%.

### 3.5. Effect of Release Agent on Concrete Surface Quality, Greasy Concrete Surface

**Figure 2:**
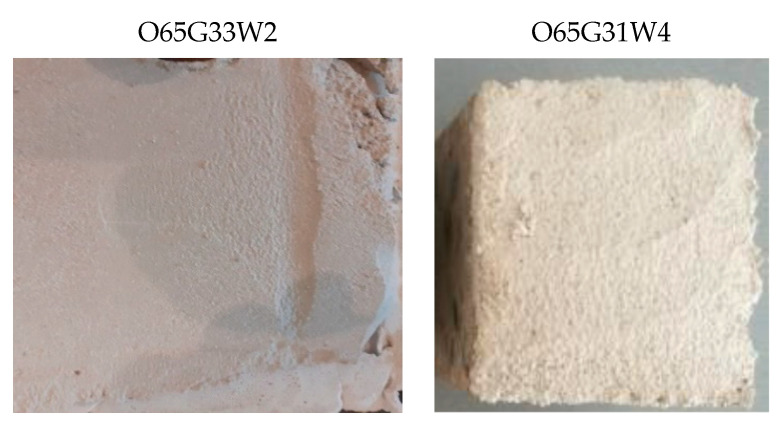

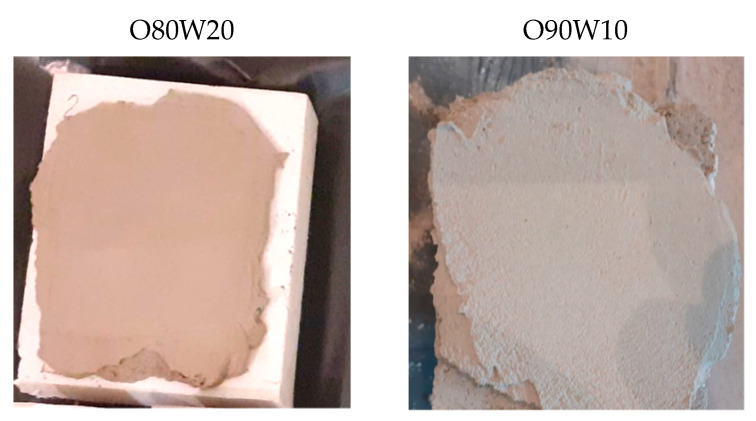
Condition of plastering on the surface of concrete specimens.

According to clause 2.3.1 of the PN-B-19305 standard [14], the greasing of the concrete surface determined by the adhesion of the plaster to the concrete according to the standard should be such that breaking of the plaster at the contact with the concrete occurs at most on one out of three tested concrete samples. Selected specimens with plastering are shown in Figure 2.

As shown in Figure 2, the oils used did not cause the greasing of the concrete surface because the plaster did not peel off the concrete surface.

### 3.6. Change of Color of Concrete Surface, Streaking (Visual Method)

**Figure 3:**
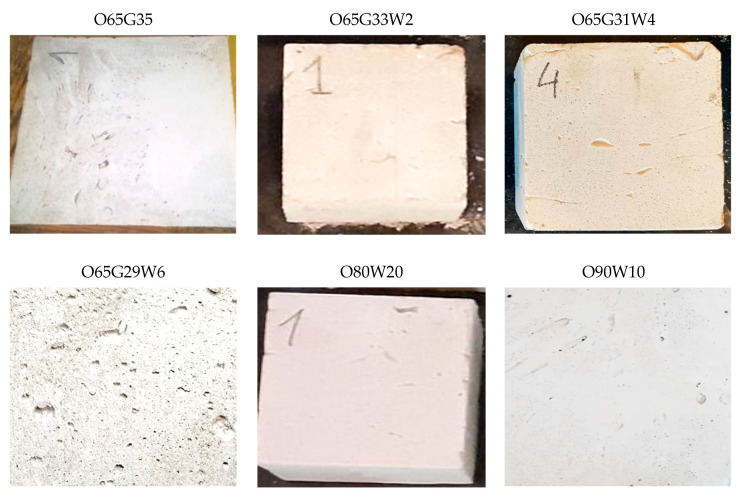
The surface condition of concrete specimens after demolding.

According to Section 2.3.2 of PN-B-19305 [14], the release agent should not cause any change in the color of the concrete (stains, crystalline efflorescence) on any of the three tested samples. Figure 3 shows the surface condition of the architectural concrete after unmolding the samples.

No staining, streaking, crystallization, or efflorescence was observed on the surface of the demolded concrete samples for all the mold release agents analyzed. Satisfactorily, the new mold release oils did not cause streaking or yellow stains on the light-colored architectural concrete, which is a very desirable characteristic.

### 3.7. Influence of Release Agent on Mould Quality

**Figure 4:**
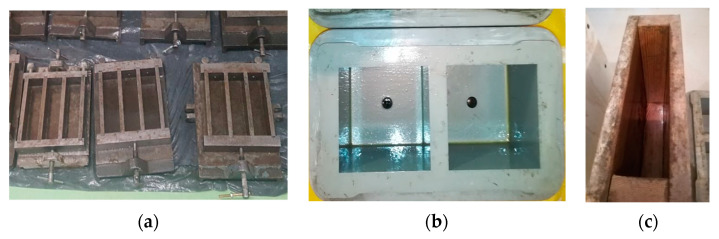
Molds covered with mold release agents: (**a**) steel molds, (**b**) plastic molds, (**c**) wood panel molds.

The molds were thoroughly cleaned, and then their walls were coated with adhesive, as shown in Figure 4.

The test was carried out in accordance with point 3.6.2.1. of the PN-B-19305 standard [14], which consisted of checking the effectiveness (adhesion) of the release agent depending on the forming time (after 1 h and 24 h of using the oil). The adhesion of concrete to the mold shall be visually verified by visually inspecting the inner surfaces of the molds after demolding the samples. No concrete adhesion to the mold surface is allowed. Concrete dust on the mold surface that can be removed with compressed air is acceptable.

We did not observe any problems with the release of samples from the molds or adhesion of concrete to the mold after either 1 h or 24 h of coating the molds with oils. The test showed that the produced release oils fulfilled their function.

### 3.8. Effect of Release Agent on the Characteristics and Durability of Concrete

#### 3.8.1. The Water Absorption Coefficient A_w_

**Figure 5:**
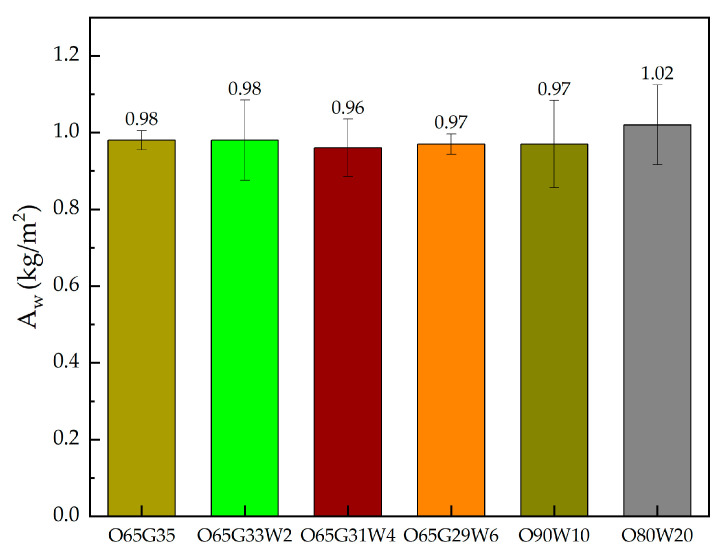
Water absorption coefficient due to capillary rise.

Figure 5 shows the results of the test carried out.

It was observed that as the amount of water in the mixture increases, the water absorption coefficient due to capillary rise increases. It differs by a maximum of 7% between the agent without water O65W35 and the highest water content O80W20. Architectural concrete obtained similar coefficient values regardless of the oil used (0.96–1.02 kg/m^2^). The concrete obtained the highest absorption coefficient at 20% water content. These values are typical for ordinary concrete; they do not deviate from the standard, which indicates the lack of adverse effect of oils on water retention in concrete.

#### 3.8.2. Wettability (CA)

**Figure 6:**
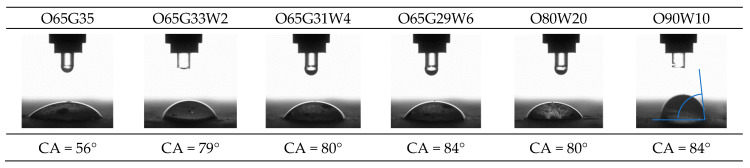
Wetting angle measurement of concrete specimens.

The contact angle (CA) analysis was performed on distilled water. Measurements were conducted at approximately 22.5 °C at the time of droplet application (Figure 6).

In the case of architectural concrete, which contains plasticizing and hydrophobizing admixtures, the contact angle was higher than ordinary concrete. It is not a disadvantage caused by the oil application but is due to the specific characteristics of this type of concrete, the admixtures used, and the lower w/c ratio. Evidence of this can be seen in the contact angle measurements, which increased by only 2–3° after oil application. Therefore, it can be concluded that the release oils did not increase the wetting angle of the analyzed concrete surfaces.

#### 3.8.3. Water Vapor Diffusion

**Figure 7:**
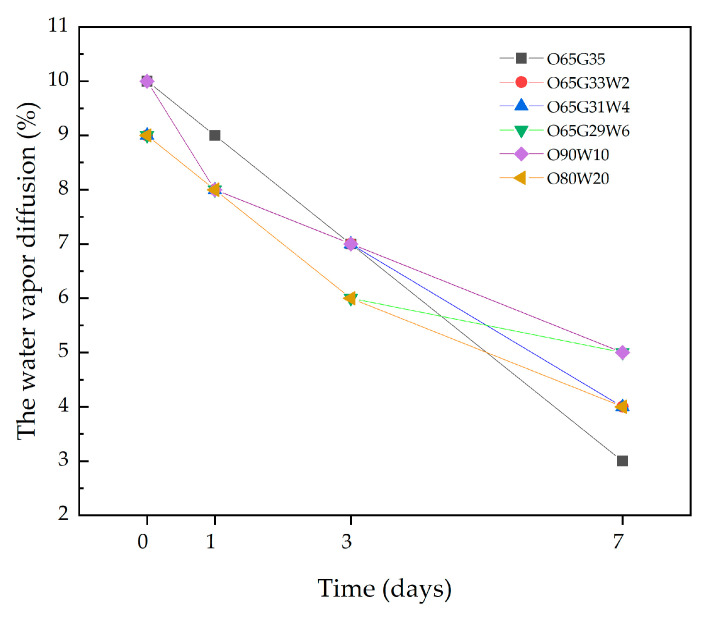
The water vapor diffusion in time.

The results of water vapor diffusion over time are shown in Figure 7.

The test showed that the tested release agents did not inhibit water vapor diffusion from the tested specimens (Figure 7). After 7 days, the moisture content was between 3 and 6%. In the case of architectural concrete, water vapor diffusion is faster, and this is due to the lower w/c of the concrete as well as the admixtures that are used in its production.

#### 3.8.4. Adhesion of Mortar to the Concrete Surface

**Figure 8:**
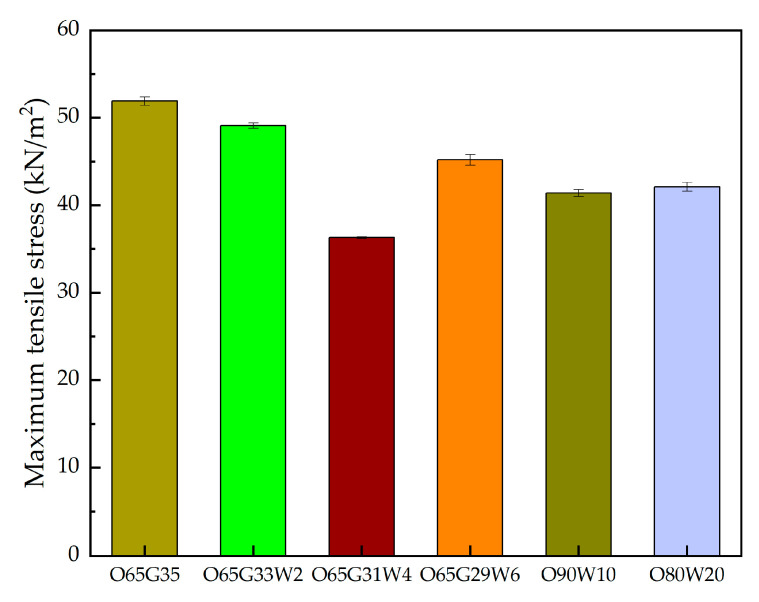
Adhesion of adhesive mortar as the maximum tensile stress induced by the peel load.

**Figure 9:**
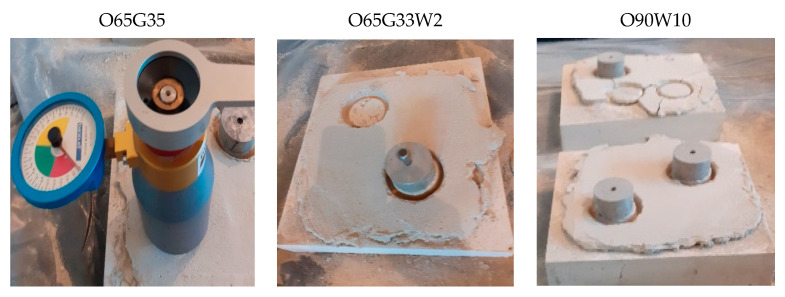
Testing the adhesion of adhesive mortar to concrete.

The study results are shown in Figure 8, while the study is illustrated in Figure 9.

The mortars showed good adhesion, ranging from 36.3 to 51.9 kN/m^2^. The plasters showed the greatest adhesion to O65G35 concrete. The antiadhesion oils did not adversely affect the adhesion of adhesive mortars to concretes.

#### 3.8.5. Effect of Release Agent on the Paintability of Concrete

**Figure 10:**
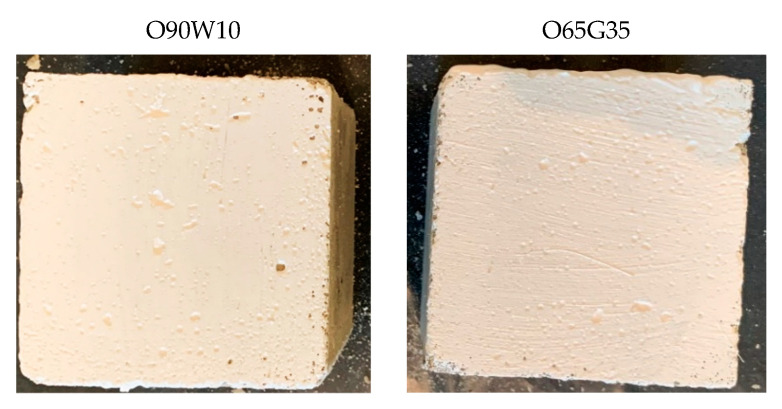

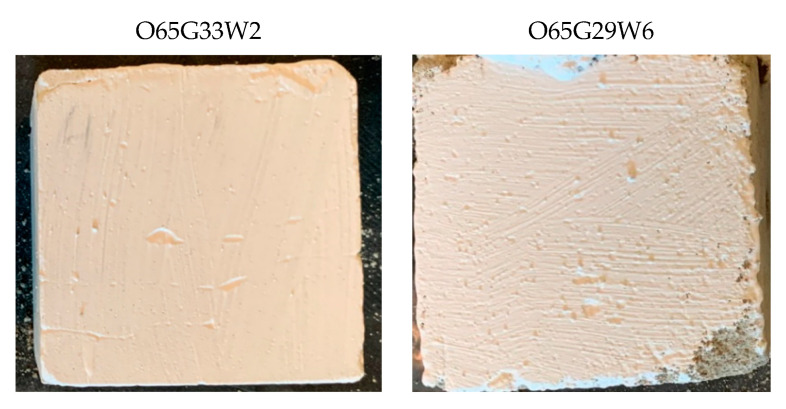
Testing the adhesion of adhesive mortar to concrete.

The condition of the samples after applying two coats of white emulsion paint is shown in Figure 10.

The plain concrete was evenly coated with paint. There were no difficulties in applying the paint, e.g., greasy spots making it impossible to cover the concrete with emulsion. Excellent paint absorbency was achieved. The generated release agents did not adversely affect the ability to coat the concrete with paint.

## 4. Discussion

Analyzing the appearance of the agents, it can be stated that in the case of O65G35, no segregation of components or precipitate was observed, even a few days after production. The color of the agent with 10% water content (O90W10) changed to a more transparent, darker color, similar to natural oils. However, a slight precipitate was observed at the bottom of the container. As the amount of water in the mixture increased, turbidity of the substance was noticed. The individual components of the new agents mixed very well together. However, as time passed, a white precipitate appeared at the bottom of the container for O90W10, O80W20, and O70W30 and a pink residue for the agents with glyceryl trioleate and water. Due to too much segregation of the mold release agent with 30% water content, further studies decided to eliminate it.

The measurements of the η coefficient showed that the agent O65G31W4 had the highest dynamic/kinematic viscosity, while the agent O90W10 had the lowest. For comparison, the kinematic viscosity of rapeseed oil methyl ester at 20 °C is 7 mm^2^/s [27]. The high viscosity makes the newly produced release agents less likely to runoff from sloping and lateral mold surfaces.

It is crucial that manufacturers or distributors provide information regarding the biodegradability, ecotoxicity, and bioaccumulation of chemical products in Section 12 of the safety data sheets for chemical products [28] prepared in EU countries under Directive 2001/58/EC [29]. Biodegradation of oils is a process induced by microbial enzymes, thanks to whereby, by transforming the chemical structure of compounds constituting the oil composition, microorganisms obtain metabolites that are incorporated into natural energy-generating and biosynthetic pathways occurring in their cells. Such property should be presented next to the functional properties in the general characteristics of release oils. The study showed that O65G31W4, O90W10, O80W20 agents experienced complete biodegradation after 21 days.

The analysis on the possibility of obtaining biodegradable release oils with planned operating properties presented by Duncan et al. [30] showed that designing the ester structure of biodegradable base oil with specific required physicochemical properties is the most crucial. Therefore, attempts are being made to produce fatty acids from natural plants, animal oils, and triglyceride fats [31,32]. Vegetable oils tested for their susceptibility to rapid biodegradation in the environment, compared to all other base oils used in the production of release agents, show the greatest biodegradability ranging between 70 and 100% [33,34], regardless of the origin and growing conditions of the plants from which they are derived. That results from the fact that they are synthesized materials by nature and used next to carbohydrates and proteins by heterotrophic organisms as a high-energy carbon and energy source. However, vegetable oils currently used as oil base release agents are oils with a modified structure, obtained either through genetic modification of plants or by chemical modification of oils [35,36,37,38].

Analyzing mortar adhesion to the concrete surface, no lack of adhesion was observed in all six studied cases. In their article, Brito et al. [39] tested the adherence of the finishing to the concrete surface after application of Vegetable oil-based Release Agents (VERA) emulsion. They presented the procedure of the test. However, they did not show specific results or observations in their work.

Satisfactorily, the new release agents did not cause staining, streaking, crystallization, or efflorescence on the light-colored architectural concrete, which is a very desirable feature. In the article Klovas and Daukšys [40], the main objective was to achieve quality changes in self-compacting concrete surfaces through different forms of application of release agents. They showed that applying an excessive amount of release agent leads to increased porosity of the concrete surface. Because of that, the BA8 vertical concrete specimens became more porous. The total flaw area concerning the total specimen area increased from 0.18% to 0.36%. Brito et al. [39] described a procedure for checking the concrete surface after application of VERA emulsion. They pointed out that the concrete changes its color after demolding; therefore, visual observation should be performed about 48 h after demolding. In [39], the authors also described a procedure for determining the porosity classification of concrete. They remarked that an essential measure of quality control in precast plants is the surface porosity of the unfinished concrete at the interface with the mold. Due to the visible uneven distribution of VERA oils on the mold surface when the majority of the emulsion water had dried, a standard test had to be developed to check the possible effect of the release agent on the surface of the concrete.

Both 1 h and 24 h after application of the release agent, there were no problems with samples being released from the molds or concrete sticking to the mold. Libessart et al. [41], in their work, have focused on concrete/formwork interface analysis. They conducted tribometer tests which demonstrated that films created using emulsion resulted in a 30% and 40% reduction in friction between the concrete/formwork interface. In the paper, Brito et al. [39] prepared samples using different water/oil ratios of VERA emulsion. A standard adhesion test was carried out for each ratio, and a friction coefficient was obtained. They observed whether the mold or the sample surface in contact with the mold was pulverized after demolding.

It was observed that as the amount of water in the mixture increases, the water absorption coefficient due to capillary rise increases. It differs by a maximum of 7% between the agent without water O65W35 and the highest water content O80W20. The highest absorption coefficient A_w_ was obtained at 20% water content. These values are typical for normal concrete and do not deviate from the standard, indicating any adverse effect of oils on water retention in concrete.

The mold release oils did not seal the structure of the concrete. All CA were lower than 90°, indicating the hydrophilicity of the concrete. The O65G35 concrete had the best wettability. In this case, the contact angle was 56°. For architectural concrete, the contact angle was much higher than for normal concretes or mortars described, for example, in the work of Barnat-Hunek et al. [42,43]. The disadvantage is not due to the use of oil but is due to the specificity of this type of concrete, the admixtures used, and the lower w/c ratio. Therefore, it can be concluded that the molding oils did not increase the wetting angle of the analyzed concrete surfaces. A different approach was taken in their work by Izarra et al. [44]. They focused on producing a release agent designed to create a hydrophobic layer on the surface of the concrete. The produced release agent by Izarra et al. [44] containing 3 wt% of MTEOS-2.5 (2.5—the molar ratio between the precursors methyltriethoxysilane (MTES) and tetraethyl orthosilicate (TEOS)) helped to produce mortar samples with contact angles greater than 145° with a good distribution of the release agent on the mortar surface. Song et al. [45] also examined the CA of commercially available release agents in their work. However, they investigated the contact angle not on the surface of the material but on the surface of the mold. In their work, they were concerned with release agents that, once applied, would allow the mold to be used repeatedly.

Water vapor diffusion is the movement of molecules in a mixture of gases to equalize the vapor concentration. Diffusion allows water vapor to pass through partitions by balancing the partial pressure prevailing on both sides of the partition. The water vapor diffusion test was conducted to see if the new release oils had a negative effect on the flow of water vapor through the concrete specimen. The test showed that the tested release agents did not inhibit water vapor diffusion from the test specimens (Figure 8). The moisture content after 7 days for all concretes was between 3 and 5%.

The mortars showed good adhesion on which the new formulations were used, ranging from 36.3 to 51.9 kN/m^2^. The plasters showed the highest adhesion to concrete for which O65G35 oil was used in the preparation. The release oils did not adversely affect the adhesion of the mortars to the concretes.

The architectural concrete was evenly covered with paint. There were no difficulties in applying the paint, e.g., greasy spots that made it impossible to cover the concrete with emulsion. The release agent did not negatively affect the ability to coat the concrete with paint.

## 5. Conclusions

Studies have shown that higher fatty acids derived from vegetable oils can be used as release oil for steel and plastic molds used to produce light-colored architectural concrete elements, meeting the normative requirements for this type of oil. Formulations have good service properties except for a white precipitate that can be filtered out as needed.

In addition, the good rheological properties, resulting from the relatively low viscosity of the oil, have a beneficial effect on the use-values, as it can be applied to steel mold surfaces by typical methods such as dipping, brushing, or spraying, without the need for dilution. Agents with no or 2–4% water content are characterized by a low freezing point (from −14 °C to −7 °C).

The newly developed release agents do not seal the concrete, allow moisture to migrate freely, no greasy film is formed on the concrete surface, the adhesive mortar shows good adhesion to the substrate. The high biodegradability of the new release agents was demonstrated, as they are made exclusively from natural substances.

Thus, the suitability of the selected molding oil for construction applications in concrete technology was confirmed. The best results were obtained with O65G35 and O90W10 mixtures.

## Figures and Tables

**Table 1:** Results of viscosity and density testing of mold release agents.

	O65G35	O65G33W2	O65G31W4	O65G29W6	O90W10	O80W20
Dynamic viscosity/(Pa·s)	0.025	0.032	0.037	0.035	0.022	0.031
Kinematic viscosity/(mm^2^/s)	25.30	31.97	33.56	31.90	21.67	27.88
Density/(g/cm^3^)	1.0	1.0	1.1	1.1	1.0	1.1

**Table 2:** Storage temperature of liquid samples.

	O65G35	O65G33W2	O65G31W4	O65G29W6	O90W10	O80W20
Temperature at which freezing of the agents occurred/°C	−15	−9	−7	−5	0	0

**Table 3:** Biodegradability test results.

	O65G35	O65G33W2	O65G31W4	O65G29W6	O90W10	O80W20
Biodegradability:						
after 7 days,%	55	55	56	56	60	60
after 21 days,%	98	99	100	98	100	100

## Data Availability

Not applicable.
